# Supplementary material for: Is there an indication for simultaneous bilateral knee arthroplasty in morbidly obese patients? Should the patients’ request for simultaneous operation be considered?
Source: PLoS One. 2023 Oct 25;18(10):e0287021. doi: 10.1371/journal.pone.0287021 (PMC10599534; doi:10.1371/journal.pone.0287021)
Supplement: Attachment — Submitted filename: Response to the reviewer II.docx [file pone.0287021.s001.docx]

Response to the reviewers

**Reviewer #1**: This is an interesting study comparing simultaneously TKA in morbidly obese patients and obese patients.

I propose publication of the present study after some minor revisions

In the abstract there is a typo in the section materials and methods (not materyals).

**Re:The typo was corrected: Materials and methods.**

Did the surgeon use tranexamic acid?

**Re:Tranexamic acid was not used in the patients.**

All the implants were cruciate retaining (CR). What about the stability in the joint especially in the MO group? Any comment from the authors?

**Re: Joint instability did not develop in morbidly obese patients due to the use of cruciate retaining (CR) implants. In terms of stability, we believe that cruciate retaining implants can be used safely in morbidly obese patients.**

**Reviewer #2:** A privilege to evaluate the study. Thank you for the opportunity to read the article and get to know the work. Study with a very relevant and interesting topic in knee surgery. More and more obese and super obese patients require knee arthroplasty. Study with a large number of patients and well designed and performed.

**Reviewer # 3 :** Thank you for submitting your manuscript to PLOS ONE for consideration.

The authors report their experience in bilateral total knee arthroplasty among patients with Morbid Obesity, and compare this to the outcomes/adverse events in a cohort of non-morbidly obese patients treated with single anesthetic bilateral total knee arthroplasty.

The study observations are not surprising, given the general considerations for increased medical risks among patients with morbid obesity compared with other patients undergoing total knee arthroplasty surgery.

The question that will arise with the comparison that the authors have selected, is whether the rates of adverse events in the morbidly obese cohort are unique to the single anesthetic bilateral TKA surgery, or whether this reflects the risk of adverse events among morbidly obese patients who are undergoing elective primary total knee arthroplasty (unilateral, staged bilateral, or bilateral).

**Re:Adverse events encountered in the morbidly obese group are not specific to bilateral knee replacement surgery performed under single anesthesia, but are also encountered in unilateral or staged bilateral knee surgery. However, morbid obesity paves the way for the development of adverse effectsin bilateral knee surgery performed with single anesthesia due to both high BMI and accompanying comorbidities.**

The reviewer suggests that a more appropriate observational study would be to compare the rates of complications among morbidly obese patients undergoing single anesthetic bilateral total knee arthroplasty with cohorts that were treated with unilateral TKA (with the second TKA not performed), or with staged bilateral TKA (second TKA performed at least 3 months after the initial procedure).

**Re: Yes. We plan to conduct this study. in the future and we are currently in the preparation process.**

The authors should ideally note the frequency in which patients who were treated with morbid obesity had this as risk factor as a solitary condition, as well as the proportion of patients who had concurrent medical diagnoses (diabetes, tobacco use, malnutrition, ischemic cardiac disease/congestive heart failure, pulmonary disease---including sleep apnea, etc). It is also important to understand the frequency of lymphedema among the patients who had wound complications and/or in the cohort of morbidly obese patients undergoing surgery.

The reviewer will presume that the morbidly obese patients who were offered a single anesthetic bilateral TKA were "otherwise healthy," but it is not clear from the manuscript whether this is true,or not.

**Re:We apologize. Comorbidities are very important. We actually included comorbiditie in the study. We encountered an error that we did not understand. Comorbidities have been added to the statistical analysis table (Table 1).**

**Re:Mild lymphedema was observed in two patients in the morbidly obese group and in three patients in the obese group.No treatment was planned for lymphedema.**

Since venous thromboembolism is among the adverse events reported, it is important to understand what the VTE prophylaxis was for these patients, and whether it was similar or different from the cohort that this patient group is being compared to.

**Re:The patients were given antithrombotic stockings together with an anticoagulant (enoxaparin 1x0.4-0.8ml) one day before the operation. Anticoagulant prophylaxis was administered up to four weeks post op. Anticoagulant prophylaxis was applied at higher doses in the morbidly obese group (2x 0.4ml) than in the obese group (1x 0.4 ml).**

Othersuggestions/considerations

Abstract:“This study aimedto analyze the feasibility of ….”

Suggest: This study aimed to analyze the safety of…..”

**Re: Changed to‘’This study aimed to analyze the safety of…..”**

Page 4: The study indicates that the patients received enoxaparin one day before the operation. Did the patients receive any additional thromboprophylaxis beyond this time point? How long was treatment continued after the operation? (2 weeks, 4 weeks, 6 weeks?)

**Re:The patients did not receive any additional thromboprophylaxis other than antithrombotic stockings. Anticoagulant prophylaxis was administered up to four weeks postop.**

Page 4: The patients received cefazolin (1 gm x 3). Is this dose appropriate for patients with a BMI > 40 kg/m2. Were antibiotics re-dosed before the start of the 2nd TKA, or was a single antibiotic dose given for the entire procedure?

**Re: Prophylactic antibiotics were administered at the same doses in morbidly obese and obese patients (Cefazolin 1 gm x 3). Antibiotic doses were not increased in morbidly obese patients in order not to adversely affect renal functions. No additional doses of antibiotics were needed before the second operation.**

The authors are reporting on a group of patients with a BMI > 40 kg/m2. What was the mean and median BMI in this cohort? What proportion of patients had a BMI > 45 kg/m2. What proportion had a BMI > 50 kg/m2? Could this potentially affect outcomes?

**Re: Mean BMI was 44.8 kg/m^2^ in morbidly obese patients and 35.2 kg/m^2^ in obese patients. BMI index was 40-45 kg/m^2^ in 58 of the morbidly obese patients and 45-50 kg/m^2^ in the remaining 7 patients.None of the patients had BMI exceeding 50 kg/m^2^. As BMI increases, it can negatively affect the results.**

Page 10: Discussion of limitations. The authors report a limitation in information available on this procedure in morbidly obese patients as a study limitation. The study limitation discussion should focus on the limitations of this study.

Examples of limitations could include: The retrospective nature of the study may not account for selection bias among patients who were included in the study. The indications for bilateral TKA surgery in this patient cohort are not specified. The outcomes of unilateral TKA (staged bilateral TKA) are not reported/considered. The scope of medical comorbidities are not accounted for, so it is not clear whether morbid obesity was the main factor that contributed to a higher risk for adverse events, or if other concurrent comorbidities were stronger risk factors---or whether concurrent disease may have amplified the rates of adverse events.

**Re:All patients with severe knee arthrosis (Grade 4) were included in the study.Patients who underwent unilateral TKA were excluded from the study, therefore, the results ofpatients who underwent unilateral TKA were not reported in our study.**

**Adverse events in morbidly obese patients should not be attributed to bilateral TKA surgery with single anesthesia. However, it should not be forgotten that morbid obesity can pave the way for these adverse events. In addition, many comorbidities of morbidly obese patients will increase the risks and adverse events due to the stress caused by bilateral surgery.**

Conclusions: The mention of the need for prospective studies would not seem justified. If the authors are reasonably convinced that bilateral TKA is not safe in morbidly obese patients, it would seem unwise to advocate for prospective studies to determine whether this finding remains true in a less biased study.

**Re: Deleted ‘’ need for prospective studies ‘’. We do not think that bilateral TKA operation with a single anesthesia in morbidly obese patients is safe. However, we anticipate that comprehensive studies on this subject will support our findings.**

It is OK for the authors to state----based on their experience----that the risks associated with SBTKA are greater among morbidly obese patients than among non morbidly obese patients. Study would be needed to define whether this risk is significantly elevated compared with the relative risk of complications associated with unilateral TKA in these subjects. Is the risk related to the increased surgical insult (2 knees instead of 1), or is this a reflection of performing TKA (single or bilateral) on this patient group----that has an increased risk of complications following arthroplasty surgery.

I sincerely wish you well in your efforts to refine the manuscript.

**Re:Risks in the morbidly obese are associated with surgical damage from bilateral TKA surgery. Since surgical damage will be much less in unilateral or staged operations in morbidly obese patients, risk development will also decrease.We believe that risk development is directly proportional to the extent of surgical damage.**

**Reviewer # 4 :**

1. Paper will need help from an editorial for help with standard English. For example, in the title alone, there are two misspellings (‘artroplasty’ and ‘simultenous’) along with an incorrect apostrophe. I won’t cover them further, but the manuscript needs significant work on this issue.

**Re: Errors in the article have been corrected.Arthroplasty and Simultaneous**

2. Abstract: The aim should point out that the comparison group is actually a non-morbidly obese cohort that remains obese, rather than the typical “normal weight” cohort comparison.

**Re: Corrected‘’comparing morbidly obese (MO) patients with obese patients.’’**

3. Abstract and Methods: How were the weights confirmed? Patient self-report only? Weighed by professionals in the clinic or hospital? Same date as the surgery?

**Re: The weight of all patients was measured by the professionals in our clinic the day before the operation.**

4. Introduction: I’m not sure what’s determining the order of the citations. They do not to be alphabetical by author last name or in manuscript mention order. Please standardize or clarify.

**Re: The order of the citations is made according to the first occurrence of the subject. No alphabetical order was made according to the surname of the author or the name of the article.**

5. General: The paper has a tendency to undercite. For example, each sentence of the first paragraph should have several citations to back up these claims.

**Re: Corrections have been made. Necessary citations were made, especially the first paragraph.**

6. General: Many abbreviations are not defined at first usage.

**Re: It was checked and the necessary corrections were made.**

7. Introduction: Last sentence of second paragraph (“However, these advantages…”) is a huge opinion statement that the authors does not back up or cite. Indeed, the authors basically answer their own stated goal for doing the study, in the introduction, without any data or stated evidence. It undermines the whole manuscript. Please delete or heavily edit. Similarly, in the following paragraph the authors state they’re trying to document advantages of SBTKA as well. They’re analyzing differences in clinical outcomes, not advantages or disadvantages.

**Re:This sentence has been deleted from the manuscript.**

**Corrected : Our retrospective study aimed to analyze the safety of SBTKA with univariate and multivariate logistic regression analyzes in MO patients**

8. Methods: The authors should note specifically that all of their selected outcomes are basically surgical complications and are not related to costs, quality of life, patient-reported outcomes, etc. that provide a more accurate and nuanced perspective of the “advantages and disadvantages” of simultaneous bilateral TKA.

**Re: We added “All outcomes in the manuscript are surgical complications and are not related to cost analysis, quality of life, patient-reported outcomes.”**

9. Methods and Results: The authors note that multivariate logistic regression techniques were done. However, most of the included adjusters appear to be surgical outcomes themselves (like operative time and debridement) rather than preoperative adjusting factors, such as smoking, baseline osteoarthritis severity, quality of life/joint pain PROMs, and comorbidities. These are vastly more important. With all we know about TKA, adjusting just by age, gender, and obesity is not enough. Nor are there more technical statistical aspects like robust standard errors, an a priori power analysis, etc.

**Re: The main focus of our study was post-op features, rather than preop features. However, age, gender and descriptive features (diabetes, ischemic heart disease, etc.) were checked for the homogeneity of the groups. These were found to be statistically insignificant.**

**In the power analysis conducted with the G*power 3.1 program, the effect size of the study groups for the time of mobilization was found to be 0.46 (*The outcomes of total knee arthroplasty in morbidly obese patients: a systematic review of the literature)* (alpha error probability=0.05). In the sample size analysis performed with a power value of 0.80, the total number of samples required to be taken was found to be 120 (at least 60 for each group).**

**In addition to descriptive statistical methods (mean, standard deviation), the Shapiro-Wilk test was used to assess the normality of data distribution.The independent t-test and the Mann Whitney U tests were used for the comparison of normally and non-normally distributed variables between paired groups. The Chi-square and Fisher's exact tests were used to compare qualitative data. To determine the factors affected by MO, multivariate logistic regression analyses were performed with the variables that were foundsignificant in the univariate tests. The results were evaluated at the significance level of p<0.05**.

10. Discussion: There is a line early in the Discussion that “Obesity is a preventable and modifiable risk factor.” No context, no citations, huge definitive opinion statement. Many obesity scholars and researchers would dispute this statement.

**Re:The sentence has been corrected and citation was added. ‘Obesity has been suggested as a modifiable risk factor[19]’.**

11. Discussion: Last sentence, limitations section: Being a single surgeon and single institution is not a strength, for it could be that the variation seen may be related to the technical skills of the surgeon, support staff, or the institution in its abilities to care equivalently for both patient cohorts. If the results hold up across multiple institutions/hospitals/surgeons, etc., the results are much stronger.

**Re: This is a different perspective. Sorry, we don't agree with this idea. The accuracy of the data to be obtained from studies performed in different centers, different surgeons, and under different conditions (pre-op, per-op, post-op) can be discussed. However,we believe the reliability of the data to be obtained in studies with a single surgeon, a single center and the same conditions is more accurate.**

12. Notably, the authors spend significant time noting significant differences in operation time and safety for morbidly obese patients, yet their Figure 1 appears to demonstrate that these factors did not in fact differ by cohort.

**Re: If Figure 1 is read carefully and accurately, it will be seen that there are statistically significant differences (hospitalization time, surgical time, mobilization time) between the cohort groups.**
